# Reliability, Feasibility, and Patient Acceptance of an Electronic Version of a Multidimensional Health Assessment Questionnaire for Routine Rheumatology Care: Validation and Patient Preference Study

**DOI:** 10.2196/15815

**Published:** 2020-05-27

**Authors:** Theodore Pincus, Isabel Castrejon, Mariam Riad, Elena Obreja, Candice Lewis, Niels Steen Krogh

**Affiliations:** 1 Rush University Medical Center Chicago, IL United States; 2 Department of Rheumatology General University Hospital Gregorio Marañón Madrid Spain; 3 ZiteLab ApS Copenhagen Denmark

**Keywords:** patient reported outcomes, health status measures, electronic version, rapid3, mdhaq

## Abstract

**Background:**

A multidimensional health assessment questionnaire (MDHAQ) that was developed primarily for routine rheumatology care has advanced clinical research concerning disease burden, disability, and mortality in rheumatic diseases. Routine Assessment of Patient Index Data 3 (RAPID3), an index within the MDHAQ, is the most widely used index to assess rheumatoid arthritis (RA) in clinical care in the United States, and it recognizes clinical status changes in all studied rheumatic diseases. MDHAQ physical function scores are far more significant in the prognosis of premature RA mortality than laboratory or imaging data. However, electronic medical records (EMRs) generally do not include patient questionnaires. An electronic MDHAQ (eMDHAQ), linked by fast healthcare interoperability resources (FIHR) to an EMR, can facilitate clinical and research advances.

**Objective:**

This study analyzed the reliability, feasibility, and patient acceptance of an eMDHAQ.

**Methods:**

Since 2006, all Rush University Medical Center rheumatology patients with all diagnoses have been asked to complete a paper MDHAQ at each routine care encounter. In April 2019, patients were invited to complete an eMDHAQ at the conclusion of the encounter. Analyses were conducted to determine the reliability of eMDHAQ versus paper MDHAQ scores, arithmetically and by intraclass correlation coefficient (ICC). The feasibility of the eMDHAQ was analyzed based on the time for patient completion. The patient preference for the electronic or paper version was analyzed through a patient paper questionnaire.

**Results:**

The 98 study patients were a typical routine rheumatology patient group. Seven paper versus eMDHAQ scores were within 2%, differences neither clinically nor statistically significant. ICCs of 0.86-0.98 also indicated good to excellent reliability. Mean eMDHAQ completion time was a feasible 8.2 minutes. The eMDHAQ was preferred by 72% of patients; preferences were similar according to age and educational level.

**Conclusions:**

The results on a paper MDHAQ versus eMDHAQ were similar. Most patients preferred an eMDHAQ.

## Introduction

A multidimensional health assessment questionnaire (MDHAQ) [1-3] has been completed by all patients at all visits to one of the authors (TP) since 1982, based on initial evidence that patient questionnaire scores for physical function are significant in the prognosis of work disability and premature death in rheumatoid arthritis (RA) [4]. Paper MDHAQ data for all patients were entered into databases (Paradox, Access) in the 1990s to provide serial flowsheets of patient scores, laboratory data, and medications, which informed clinical decisions [5]. Clinical research using MDHAQ databases has advanced knowledge concerning the prognosis of mortality [6-8], importance of socioeconomic status [7,9,10], value of methotrexate [11,12], value of low-dose glucocorticoids [13], and recognition of depression [1,14] in RA and other rheumatic diseases.

The MDHAQ is completed by all patients at several rheumatology sites, including New York University Hospital for Joint Diseases (since 2005), a private practice in Ridley Park, PA (since 2006), Rush University Medical Center (since 2006), and Liverpool Hospital in Australia (since 2013) [15]. However, the patients continue to complete a paper MDHAQ despite the use of electronic medical records (EMRs) at each of these sites, as the EMR generally has not included patient questionnaires. Databases of paper MDHAQ data from routine care have been entered into electronic research databases to recognize that disease burden in osteoarthritis is similar to RA [15], and to develop indices such as routine assessment of patient index data 3 (RAPID3) [16-18] and a fibromyalgia assessment screening tool (FAST3). RAPID3 is the most widely used RA index in the United States [19,20], and comparable to disease-specific indices to recognize changes in clinical status in patients with all other rheumatic diseases which have been studied [21-23].

An electronic MDHAQ (eMDHAQ), linked to an EMR through fast healthcare interoperability resources (FHIR), could enhance clinical care with serial flowsheets, enable remote patient completion before or between scheduled visits to report problems, and reduce costs and errors in retrospective data entry of paper versions for clinical research. This report presents analyses of the reliability, feasibility, and patient acceptance of an eMDHAQ.

## Methods

### Ethics and Consent

The Rush University Institutional Review Board waived the requirement for patient consent in the completion of patient questionnaires, as the questionnaire is a component of routine care, analogous to a laboratory test, that provides quantitative quality measures to guide clinical decisions. The database used in this study is part of the Rush University Patient-Reported Outcomes Studies approved by the Rush University Institutional Review Board, with a waiver for patient consent for retrospective data analysis (14090502-IRB02-AM03). The eMDHAQ is regarded as an extension of efforts to implement quality measures.

### Patients

Since 2006, all patients with all diagnoses seen by all clinical rheumatologists at Rush University Medical Center (14 in 2019) have been asked to complete a paper MDHAQ at each visit to provide quality measures in routine care [24]. Since the introduction of the Epic EMR at Rush in 2011, all completed MDHAQs have been scanned into the EMR as PDFs, incorporated into each patient’s encounter record.

In April 2019, all rheumatologists were asked to request that patients older than 18 years complete an eMDHAQ on an iPad at the conclusion of the encounter, indicating that the patient could decline for any reason. This study was conducted during routine care, as has been the case in all development of the MDHAQ/RAPID3 other than retrospective analyses of clinical trial results to compare RAPID3 to traditional RA indices [24]. Clinicians were not asked to collect formal records of how many patients were asked to volunteer or how many refused. An informal query indicated that most patients declined because of a need to leave the clinic.

### The MDHAQ

The paper MDHAQ was developed over 25 years as a 2-page patient self-report questionnaire, completed in 5-10 minutes, although no formal studies of time for completion have been reported. The MDHAQ includes patient self-report quantitative scores for physical function (FN), 3 visual numeric scales (VNS) for pain, patient global assessment (PATGL), and fatigue, a self-report painful joint count, termed the rheumatoid arthritis disease activity index (RADAI) [25], which is informative in many rheumatic diseases [26], a 60-symptom checklist [27,28], exercise status [29], morning stiffness, and change in status [27]. The MDHAQ queries recent patient medical history information, including possible surgery, hospitalizations, new medications, adverse medication events, changes in medications, and demographic data such as gender, ethnicity, and years of education. A long version of the MDHAQ (termed "4 page version" in paper format) for new patients includes past medical history, illnesses, allergies, family history, social history, comparable to a standard “intake” questionnaire for new patients [24].

MDHAQ scores have been developed into 4 indices. RAPID3 includes 3 scores of 0-10 within the MDHAQ for physical function, pain VNS, and PATGL VNS, which are compiled into a score of 0-30 [16,23,30,31]. FAST3 is a 0-3 cumulative index based on RADAI ≥16 (=1), symptom checklist ≥16 (=1), and pain and/or fatigue VNS ≥6 (=1) [32,33]; a score ≥2/3 for FAST3 agrees more than 80% with the polysymptomatic distress scale, which is the basis for the 2011 revised formal fibromyalgia criteria [32,33]. PSYCH3 (Psychological Index 3) includes queries for sleep quality, anxiety, and depression [1,14]; as a screening tool, it shows good agreement with the Centers for Epidemiologic Studies Depression Scale [1]. MEDI60 is based on the symptom checklist and has been used for remote electronic monitoring of adverse events and patient status without face-to-face patient visits [28].

### Databases

As noted above, all completed MDHAQs have been scanned as PDFs into the Epic EMR since its introduction at Rush University Medical Center in 2011 as a quality component of the encounter record. A proposed eMDHAQ/RAPID3 presented by Epic in 2015 lacked flowsheets to depict serially patient scores, reports of specific new symptoms on the MDHAQ checklist to facilitate a physician’s review of systems, and other features that had enhanced the efficiency of patient care in the pencil-and-paper data entry versions as early as the 1990s and early 2000s [24]. As features beyond straightforward scores could not be made available, an electronic MDHAQ was developed in 2015-16 with the FHIR interface to be compatible with the Epic EMR, which is termed ClinDat.

The ClinDat software is managed by ZiteLab, a Copenhagen information technology company that has managed a Danish rheumatology registry called DANBIO (initially designated “Danish Biologics Registry”) since 2002. DANBIO currently includes self-report data from more than 50,000 patients; these data have been analyzed in more than 200 published reports [34,35]. The ClinDat software was designed to be compliant with the Health Insurance Portability and Accountability Act (HIPAA), with direct patient entry of MDHAQ responses linked to an EMR through FHIR. Database management includes flowsheets depicting MDHAQ scores, laboratory test results and medications, and possible automated encounter reports, as was previously available with paper entry in the 1990s [5].

Relatively early adoption of ClinDat for routine patient care was anticipated; however, administrative delays have persisted to date. Therefore, in 2016, a decision was made to ask a research assistant or associate to enter selected scanned MDHAQs into ClinDat for specific research protocols. Protected health information, including name, date of birth, and medical record number, has not been entered into ClinDat pending approval by Rush University. ClinDat assigns a unique identifier to each patient; the unique ClinDat number is linked to protected health information in a local Excel spreadsheet on the Rush University server.

Research studies based on retrospective analysis of the ClinDat database were conducted at Rush University from 2016-2019. In one such study, it was found that according to RAPID3 and other MDHAQ scores, the disease burden in patients with osteoarthritis is similar to and often greater than that seen in patients with RA, contrary to traditional paradigms [15,36]. Discordance of global assessments by patients and physicians was found to be significantly associated with female gender, low socioeconomic status, and high pain scores [37]. MDHAQ/FAST3 was found to be in 80% agreement with formal fibromyalgia criteria [33]. The MDHAQ/MEDI60 symptom checklist has been used in remote electronic monitoring to recognize adverse events and their resolution with clinical improvement [28].

The content of the eMDHAQ is identical to that of the paper version; it is presented on 6 screens to minimize scrolling. For the study described in this report, the ClinDat unique identifier of each volunteer (or a new number for a few “new” patients) was entered into the tablet by office staff before the tablet was given to the patient. The patient then completed all eMDHAQ items on the tablet without any further interaction with the staff member before returning the tablet to the staff member.

The patient was then asked to complete a brief paper self-report questionnaire with 3 items. The first 2 items were VNS queries which were identical to those for pain, global assessment, and fatigue on the MDHAQ: A. “How helpful do you feel the questionnaire is to you to help communicate with your doctor?” B. “How helpful do you feel the questionnaire is to your doctor to help communicate with you?” The anchors were “0=not helpful at all” and “10=very helpful.” The third item was a simple query: “Which version do you prefer?” with 3 response options: "computer," "paper," and "doesn’t matter."

The comparison of the eMDHAQ to the paper version is regarded as a quality improvement project and is exempt from patient consent by the Rush University Institutional Review Board. The paper MDHAQ was entered into the ClinDat database at a later time.

### Data Analysis

Descriptive statistics were calculated for mean, range, and SD or proportion of patients according to demographic data and diagnosis. The paper and electronic MDHAQ scores were compared for reliability using paired t-tests for continuous variables and the McNemar test for binary variables. Reliability was also examined according to the intraclass correlation coefficient (ICC) with 95% CI; values less than 0.5 indicate poor reliability, 0.5-0.75 indicate moderate reliability, 0.75-0.9 indicate good reliability, and >0.90 indicate excellent reliability [38]. Feasibility was analyzed as the time to complete the eMDHAQ. Patient preference was analyzed according to the proportion of patients who responded that they preferred the eMDHAQ or paper MDHAQ or had no preference; the proportions were calculated for all patients, and according to age (≤65, >65 years) and formal education (<12, 12, or >12 years). The level of statistical significance was set as *P*<.05. All analyses were conducted using STATA 12.0 for Mac (StataCorp LP).

## Results

Among the 98 patients in the study, the mean age was 53.8 years (range 21.0-88.0, SD 16.6); 87 (89%) were female, 47 (48%) were white, 26 (27%) were black, and 25 (25%) were members of other ethnic groups. The patients had various ICD-10 diagnoses, which were assigned by the treating rheumatologist (Table 1). These patients appear to represent a typical cross-sectional group of patients seen in an academic rheumatology setting according to age, gender, and diagnosis.

The electronic and paper MDHAQ mean scores (SD) were almost identical (Table 2).

Differences ranged from –0.4 to 0.7 and were all within 2% of one another; no scores differed significantly, either clinically or statistically. The ICC for the symptom checklist was 0.86, indicating good reliability, and all other ICCs were greater than 0.92, indicating excellent reliability (Table 2).

None of the volunteer patients reported difficulties using the iPad. The mean time to complete the 6-screen eMDHAQ was 8.2 minutes, indicating good feasibility. For the patient self-report paper questionnaire, the mean VNS rating (0-10, where 10=very helpful) of how helpful the MDHAQ was to the patient was 8.8 (SD 1.7); the mean rating of how helpful the MDHAQ was to the physician was 8.7 (SD 1.9). Patient preferences were 72% for the electronic version versus 7% for the paper version, while 21% noted no preference. Preferences did not differ meaningfully according to age or level of education (Table 3).

**Table 1:** Demographic characteristics and diagnoses of the patients included in the study (N=98).

Characteristic			Value
Age (years), mean (range, SD)			53.8 (21.0-88.0, 16.6)
Female gender, n (%)			87 (89)
**Ethnicity, n (%)**			
	White	47 (48)	
	Black	26 (27)	
	Other	25 (25)	
Education level (years), mean (range)			14.6 (4-20)
**Diagnosis, n (%)**			
	Rheumatoid Arthritis	18 (18)	
	Osteoarthritis	15 (15)	
	Systemic lupus erythematosus	11 (11)	
	Osteoporosis	6 (6)	
	Fibromyalgia	5 (5)	
	Spondyloarthropathies	3 (3)	
	Vasculitis	3 (3)	
	Other	37 (38)	

**Table 2:** Scores and test-retest reliability of patient-reported measures on the MDHAQ in paper versus electronic format for patients seen in routine care (N=98).

Paper iPad Diff. (95% CI) ICC (95% CI)	Paper MDHAQ^a^ score, mean (SD)	Electronic MDHAQ score, mean (SD)	Difference (95% CI)	ICC^b^ (95% CI)
Physical function (0-10)	1.8 (1.6)	1.8 (1.6)	0.003 (–0.4 to 0.5)	0.97 (0.97 to 0.98)
Pain VNS^c^ (0-10)	4.7 (3.1)	4.9 (3.2)	–0.1 (–1.0 to 0.7)	0.95 (0.92 to 0.97)
PATGL^d^ VNS (0-10)	4.2 (2.7)	4.4 (2.8)	–0.2 (–1.0 to –0.6)	0.96 (0.95 to 0.98)
Fatigue VNS (0-10)	3.3 (3.0)	3.5 (3.1)	–0.1 (–1.0 to 0.7)	0.95 (0.93 to 0.97)
RAPID3^e^ (0-30)	10.8 (7.0)	11.2 (6.9)	–0.4 (–2.3 to 1.6)	0.98 (0.97 to 0.99)
60- Symptom checklist (0-60)	9.9 (8.8)	9.7 (8.6)	0.3 (–2.1 to 2.7)	0.86 (0.79 to 0.91)
Self-report RADAI^f^ painful joint count (0-48)	10.5 (10.1)	9.7 (9.4)	0.7 (–2.0 to 3.5)	0.92 (0.88 to 0.95)

**^a^**MDHAQ: multidimensional health assessment questionnaire.

^b^ICC: intraclass correlation coefficient.

^c^VNS: visual numeric scale.

^d^PATGL: patient global estimate.

^e^RAPID3: Routine Assessment of Patient Index Data 3.

^f^RADAI: Rheumatoid Arthritis Disease Activity Index.

**Table 3:** Preferred version of the MDHAQ according to patient age and education level, n (%).

Patient demographic		Paper MDHAQ^a^		eMDHAQ		No preference	
**Age (years)**							
	≤65 (n=69)		5 (7)		50 (72)		15 (21)
	>65 (n=28)		2 (7)		19 (71)		6 (22)
**Education (years)**							
	<12 (n=9)		1 (11)		7 (78)		1 (11)
	12 (n=18)		1 (6)		13 (72)		4 (22)
	>12 (n=68)		4 (6)		48 (70)		16 (24)
Total (N=95)		6 (6)		68 (72)		21 (21)	

**^a^**MDHAQ: multidimensional health assessment questionnaire.

## Discussion

The eMDHAQ performed similarly to the traditional paper MDHAQ. All ICCs were greater than 0.86, indicating good to excellent reliability of the 2 versions; these values are as high as those seen in most comparisons in clinical medicine. For example, an earlier report compared electronic and paper versions of RA core data set measures [38,39], indicating ICCs for a swollen joint count of 0.78 and for a tender joint count of 0.83; ICCs for RA indices which include a formal joint count were 0.85 for Disease Activity Score 28 and 0.89 for the Clinical Disease Activity Index. In an earlier study, the ICC for the self-report physical function was 0.96, that for pain was 0.88, that for patient global assessment was 0.78, and that for RAPID3 was 0.90 [38], compared to 0.97, 0.95, and 0.96, and 0.98, respectively, in the present study (Table 2). The higher ICCs in the present study may be explained in part by the 5-7 day interval between measures in the earlier study compared to 1-2 hours in the present study; also, patients seen at Rush University Medical Center have extensive experience with the MDHAQ. The previous data are presented to document that the ICCs of the measures reported by patients were somewhat higher than those obtained by physicians.

The mean time required to complete the eMDHAQ was 8.2 minutes, which appears to be acceptable. Formal studies have not been reported for the time to complete a paper MDHAQ, although informal observations over more than 30 years suggest that 5-10 minutes are required. In general, <10 minutes appears to be acceptable, although some patients likely will require more time, as is the case with the paper version.

Approximately two-thirds of patients expressed a preference for the electronic version of the MDHAQ. However, no specific information was collected concerning the number of patients who declined to complete an eMDHAQ in the routine care setting. It is reassuring that no meaningful differences in preference or reliability were seen according to age or education level, although it is anticipated that problems with completion of an eMDHAQ will be more likely in older or less educated patients, as has been seen with the paper MDHAQ; thus, differences may emerge with larger numbers of patients. However, several reports indicating poor clinical status associated with low formal education level [7,9,40] required completion of a patient questionnaire by the patients analyzed in the studies. A small fraction of patients preferred the paper version, and it is anticipated that a paper version will be offered to some patients even after the eMDHAQ is incorporated into routine care.

Clinical decisions in rheumatology patients are based more on information from a patient history than in many chronic diseases in which decisions are dominated by a “gold standard” biomarker, such as blood pressure in hypertension or serum glucose in diabetes [41]. A patient self-report questionnaire depicts components of a “subjective” [42] patient medical history as structured, quantitative, standard, protocol-driven, data which meet criteria for the scientific method [43,44]. Physical function reported on a patient questionnaire is far more significant in the prognosis of premature mortality in RA than any laboratory or imaging data [4,6,45] and is as significant as smoking to predict mortality in a nondiseased elderly population [46].

The value of RAPID3 in rheumatology care [19,20,22] is attributable in part to its capacity to depict change in clinical status in all rheumatic diseases studied to date [21,22], while the patient does almost all the work. Nonetheless, availability of additional MDHAQ scales provide a clinician with considerably more information than only RAPID3, for fatigue [47], RADAI self-report painful joint count [26], adverse events of medications and their resolution [28]. Traditional medical history queries on the MDHAQ save time for patients and doctors [31].

Quantitative RAPID3 scores are highest in patients with fibromyalgia [48], which is seen as a comorbid condition in 20–40% of patients with RA, OA, and many rheumatic diagnoses [49-51]. Clinical improvement is far less likely in patients with comorbid fibromyalgia with any diagnosis than in patients who have this diagnosis and no evidence of fibromyalgia. A further MDHAQ index, FAST3 (fibromyalgia assessment screening tool) may be used to screen for fibromyalgia [32,33], and potentially explain unchanged RAPID3 scores in RA patients with comorbid (secondary) fibromyalgia. FAST3 includes the RADAI self-report painful joint count and symptom checklist [32,33], and therefore is not available when only RAPID3 is queried.

The eMDHAQ was developed using FHIR as an internal format to save data in a Zope object database, with capacity to interface with any EMR in the management of individual patients. Further description of such integration is beyond the scope of the present study, which was focused on reliability of the eMDHAQ and its acceptability to patients. Establishment of these features is regarded as an important future stage in development of an eMDHAQ for implementation in routine clinical care.

This study has several important limitations. First, all patients completed the paper MDHAQ as a usual component of routine care before seeing the rheumatologist, and they completed the eMDHAQ at the conclusion of the visit. Ideally, half might have completed the eMDHAQ initially in a formal study, but such a study would have presented increased costs, logistic complexities, and an extra burden to patients in a setting at which all patients complete an MDHAQ routinely. Second, patient recall of previous completion of the MDHAQ approximately 30-90 minutes earlier could have influenced the second recording. Third, neither the number of patients who declined to participate nor the reasons for declining were recorded in a routine care setting; in general, clinicians reported informally that patients who declined participation noted other scheduled activities, but some patients may have wished to avoid the computer version. Fourth, patient preference for the electronic version may be attributed in part to a “Hawthorne effect,” with further attention from a research professional and a new approach. At the same time, many patients had completed many paper MDHAQs previously, and a bias to favor the familiar paper version could have been present. The eMDHAQ appeared acceptable to most patients, although provision for a paper version for some patients is anticipated in clinical implementation of an eMDHAQ for routine care.

The analyses reported here focused on cross-sectional reliability, which is regarded by institutional information technology professionals and developers as a prerequisite for further work toward use of an eMDHAQ in routine care. Several adjustments to the workflow will be needed to implement an eMDHAQ in routine care; these details remain to be addressed in the next phases of development. Further programming and collaboration with EMR vendors for exchange of eMDHAQ data with the EMR, using FHIR available within the software, is anticipated.

An eMDHAQ presents advantages to allow completion at home before scheduled visits, rather than in the waiting area, and remotely between visits to document status when patients report disease flares or adverse medication events. Data entered by patients can be transferred to the hospital EMR using FHIR and can be made available on the physician’s computer screen. The ClinDat software for the eMDHAQ described in this report may partially overcome the problem of incompatibility of different EMRs [52] and the costs of data entry to analyze long-term outcomes of multiple rheumatic diseases in routine care, as it may be possible to facilitate pooling of deidentified data from multiple settings to establish a cost-effective multicenter database to assess clinical status and responses to therapies [34,53,54]. The preliminary results presented in this report suggest further steps toward implementation of an eMDHAQ for routine care.

## Abbreviations

EMR: electronic medical record
eMDHAQ: electronic multidimensional health assessment questionnaire
FAST3: Fibromyalgia Assessment Screening Tool 3
FHIR: fast healthcare interoperability resources
ICC: intraclass correlation coefficient
MDHAQ: multidimensional health assessment questionnaire
PATGL: patient global assessment
RA: rheumatoid arthritis
RADAI: Rheumatoid Arthritis Disease Activity Index
RAPID3: Routine Assessment of Patient Index Data 3
VNS: visual numeric scale
